# Effectiveness of Conventional Periodontal Treatment With Tetracycline Fiber Versus Minocycline Gel Application Subgingivally in Periodontitis Patients

**DOI:** 10.7759/cureus.55167

**Published:** 2024-02-28

**Authors:** Siti Lailatul Akmar Zainuddin, Norjehan Latib, Haslina Taib, Basaruddin Ahmad, Muhammad Annurdin Sabarudin, Wan Majdiah Wan Mohamad

**Affiliations:** 1 School of Dental Sciences, Universiti Sains Malaysia, Kelantan, MYS; 2 Unit of Periodontics, School of Dental Sciences, Universiti Sains Malaysia, Kelantan, MYS; 3 Dental Public Health, School of Dental Sciences, Universiti Sains Malaysia, Kelantan, MYS; 4 Periodontology, Universiti Sains Islam Malaysia, Kuala Lumpur, MYS

**Keywords:** non-surgical periodontal therapy, local antibiotics, minocycline, tetracycline, periodontitis

## Abstract

Background: Locally delivered antibiotics are adjunctive therapies for the selective removal or inhibition of pathogenic microbes in combination with scaling and root planing (SRP) for the management of periodontitis.

Objective: The primary objective of this study was to evaluate the effectiveness of tetracycline fibers against minocycline gel when used as local drug delivery in conjunction with SRP for treating periodontitis.

Methods and materials: This is a pilot randomized open single, blinded trial study comparing three treatment modalities: SRP with topical tetracycline fibers (SRP+T), SRP with topical minocycline HCL 2% gel (SRP+M), and SRP only as a control group. Probing pocket depth (PPD), clinical attachment loss (CAL), and bleeding on probing (BOP) percentages were recorded at baseline, one month, and at the end of three months. The data were subjected to analysis using IBM Corp. Released 2019. IBM SPSS Statistics for Windows, Version 26.0. Armonk, NY: IBM Corp. Repeated measures ANOVA was used to compare the clinical outcomes between the three treatment groups, accounting for the repeated measurements at baseline, one month, and three months. A p-value less than 0.05 at a 95% confidence interval was deemed statistically significant.

Results: There were statistically significant changes within the groups in all the clinical parameters, including pocket depth, clinical attachment loss, and bleeding on probing score, at different time intervals, with the greatest mean pocket depth changes seen in the tetracycline group after one month (mean changes = 1.4 mm, P < 0.001) and over three months (mean changes = 1.79 mm, p < 0.001). For clinical attachment loss, after one month, the highest improvement in clinical level was seen in the minocycline group (mean changes = 0.7mm, p < 0.05), and the overall improvement was seen in the control group (mean changes = 1.1mm, p < 0.05). The minocycline group showed greater mean changes in bleeding on probing percentage, with the greatest changes after one month (mean changes = 19.34%, p < 0.001) and over three months (mean changes = 26.42%, p <0.001). However, there was no significant difference between the groups.

Conclusion: Locally delivered tetracycline and minocycline gel are effective as adjuncts to SRP and may improve the healing outcome in the management of periodontitis.

## Introduction

Periodontitis is a chronic inflammation of periodontal tissue resulting from chronic exposure to bacterial-harvesting dental plaque biofilms such as *Aggregatibacter actinomycetemcomitans*, *Prevotella intermedia*, *Porphyromonas gingivalis*, and other microorganisms [1]. The gold standard in the treatment of periodontitis is a mechanical debridement of the periodontal pocket by scaling and root planing (SRP) [2]. This approach has its own limitations, mainly related to the inability to access deep periodontal pockets and furcations to eliminate these periodontopathogens [3,4]. Periodontal flora has a vital role in the initiation and progression of periodontal disease, and its diverse range of microorganisms renders the use of different antimicrobials in the treatment of periodontitis an adjunctive therapy to mechanical debridement [5].

Tetracycline is a broad-spectrum antibiotic group of drugs active against a wide range of gram-positive and gram-negative bacteria, whereas minocycline (7-dimethylamino-6-dimethyl-6-deoxytetracycline) is a second-generation, semi-synthetic tetracycline analogue [6,7]. The earlier studies of using tetracycline as an adjunct to SRP reported that tetracyclines possess several unique non-antibacterial characteristics that may contribute to their efficacy in periodontal therapy, which include collagenase inhibition, inhibition of neutrophil chemotaxis, anti-inflammatory effects, anti-apoptosis, microbial attachment inhibition, and root surface conditioning [7,8]. Additionally, apart from having immunomodulatory properties, it was established that minocycline stimulates osteoblasts and has an important role in enhancing periodontal healing [9].

The sole reliance on SRP may not be adequate for eradicating bacteria from the periodontal pocket, especially in hard-to-reach regions. Consequently, systemic and local antibiotics have served as supplementary agents in the treatment of periodontal disease for an extended period of time [10]. Based on a previous narrative review, local antimicrobial applications adjunctively to SRP demonstrated additional clinical benefits. This is especially crucial due to the potential complications associated with the prolonged and repeated use of systemic antibiotics. Such complications may include superimposed secondary infections, the emergence of resistant strains, and potential challenges in maintaining patient adherence. Other potential advantages include minimal side effects associated with local antibiotic use and favorable patient adherence as compared to systemic therapy [11]. By restricting the antibiotic agent to the periodontal pocket and minimizing systemic absorption, local administration offers a way to avoid many side effects typically linked to systemic antibiotic therapy [12].

Various studies have revealed that local drug delivery into the periodontal pockets can provide higher therapeutic concentrations of the antibiotic compared to systemic administration [11,12]. The use of sustained-release formulations to deliver antibacterials to periodontal pockets has recently gained interest. These products provide a long-term and effective treatment at the site of infection at much smaller doses. Biodegradable polymers are extensively employed in periodontal drug delivery devices because of their abundant source, lack of toxicity, and high tissue compatibility [13]. Local periodontal adjunctive therapies can be generally classified based on the duration of their availability within the periodontal pocket as nonsustained-, sustained-, or controlled-release delivery. The highly concentrated active agents are expected to be retained within the periodontal pockets to eradicate the causative bacteria, impede the formation of subgingival dental biofilm, or aid in the early resolution of inflammation, and promote wound healing, with controlled-release devices having a longer duration compared with sustained-release devices [14]. The last 20 years have seen the emergence of a range of adjunctive antimicrobial regimens designed to aid the mechanical methods of dealing with subgingival plaque [12].

The administration of antibiotics directly to the subgingival area (within the periodontal pocket) in conjunction with SRP has proven to be more effective than SRP alone. A stable and sustained-action formulation of minocycline gel has been developed specifically for subgingival use. Extensively evaluated in clinical trials conducted in Japan, this formulation contains 2% minocycline, a concentration chosen based on clinical, bacteriological, and gingival crevicular criteria [12]. A prior study by Abbas et al. (2016) also showed that repeated subgingival application of minocycline ointment during periodontitis treatment resulted in significant improvement following subgingival instrumentation [15]. Additionally, minocycline possesses widespread bacteriostatic properties and acts slowly by binding to the tooth surface, inhibiting the activity of collagenase at low concentrations, thus preventing periodontal tissue destruction in periodontitis [15].

Therefore, this study aimed to determine the efficacy of the local application of tetracycline fibers (Periodontal Plus AB®) and minocycline HCL 2% gel as adjuncts to SRP.

## Materials and methods

**Figure 1:**
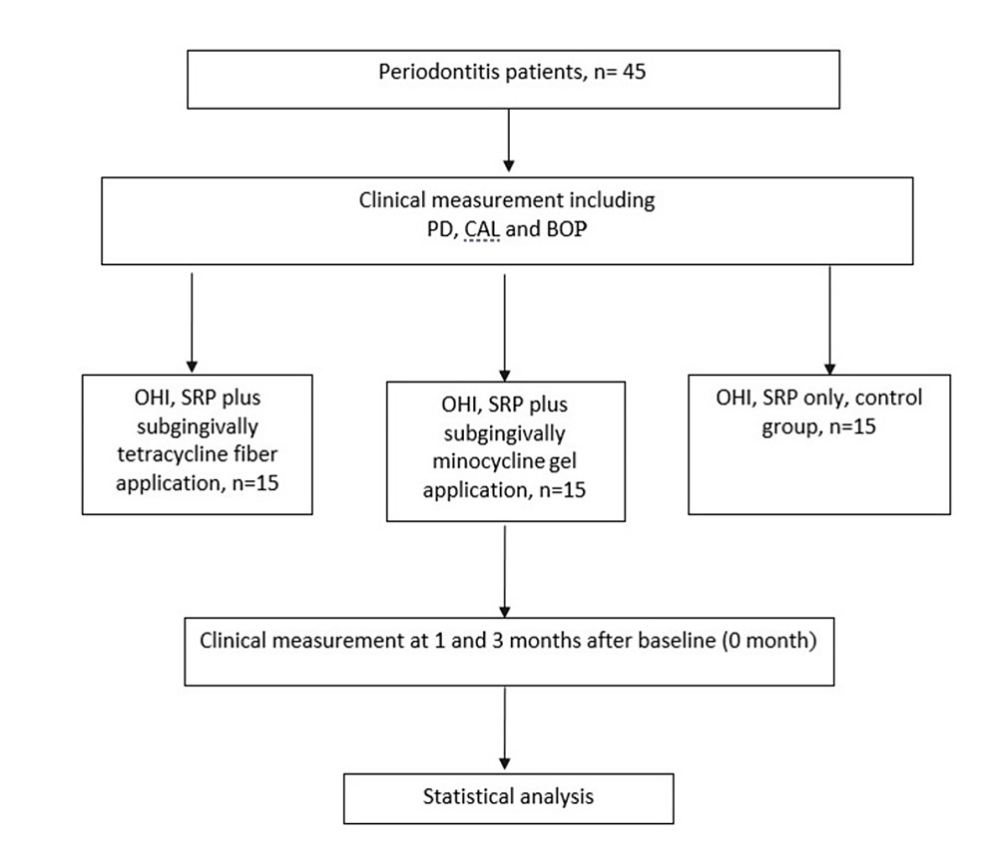
Flowchart of the study protocol

This is a pilot randomized, open, single-blinded trial study conducted in the Periodontic Clinic, Hospital Universiti Sains Malaysia, Kelantan, using the blocked randomization method and sealed envelopes. A total of 45 patients with a mean age of 52 ± 10.6 years had participated and were randomly assigned equally into three groups of three treatment modalities, which were SRP with topical tetracycline, Periodontal Plus AB® (SRP+T), SRP with topical minocycline HCL 2% gel (SRP+M), and SRP only as a control group. The inclusion criteria include patients who were diagnosed with periodontitis, did not receive any periodontal treatment, or consumed antibiotics three months before the commencement of the study. Patients who were pregnant or intending to get pregnant during the study period, diagnosed with bone diseases, and in an immunocompromised state were excluded. The recruited patients were randomly allocated to one of the groups. The reproducibility of the probing for clinical parameters was done to obtain information on the error of these measurements for an adequate interpretation of the results. All clinical measurements were performed by a single calibrated examiner. The intra-examiner reliability and reproducibility of measurements of pocket depth and clinical attachment loss had been assessed prior to the beginning of the study. Repeated measurements had been performed on a total of five chronic periodontal patients, with at least two-hour intervals between each examination. All the measured values were analyzed using the intra-class correlation coefficient (ICC) test and resulted in agreement with Chronbach’s alpha of 0.78. All 45 patients received concurrent conventional treatment consisting of scaling and root planing using ultrasonic and hand curretes until tactilely free of deposit under local anesthesia for all ≥ 5mm pockets at baseline and reviewed after one month and three months. Fifteen patients (first group) were treated with tetracycline fiber subgingivally; the other fifteen patients (second group) were treated with minocycline hydrochloride microsphere gel immediately after the scaling and root planing; and the last fifteen patients (control groups) only received scaling and root planing at baseline. All groups reviewed at one month and three months and received oral hygiene instructions (plaque control instruction) and oral prophylaxis of the teeth at review visits as necessary. The study protocol was approved by the Human Research Ethics and Committee, Universiti Sains Malaysia (USMKK/PPP/JEPeM/1405205). These procedures are summarized in Figure 1.

Data analysis

The analysis utilized IBM Corp. Released 2019. IBM SPSS Statistics for Windows, Version 26.0. Armonk, NY: IBM Corp. Prior to analysis, the data underwent thorough checking and cleaning. Descriptive analyses were then reported, presenting mean (SD) for continuous variables and frequency and percentage (%) for categorical variables. As they were normally distributed, the data was analyzed using parametric tests. Repeated measures ANOVA was used to compare the clinical outcomes between the three treatment groups, accounting for the repeated measurements at baseline, one month, and three months. The significant level for all analyses was set at 5%.

## Results

There were 24 (53.3%) male and 21 (46.7%) female subjects involved in the trial. Malays were dominant in all groups (73.3%), which reflected the composition of Malay ethnicity in Kelantan (Table 1).

**Table 1:** Demographic characteristics in all groups of the study subjects (n=45).

Variables		Frequency, n (%)		
		Tetracycline group (n = 15)	Minocycline group (n = 15)	Control group (n = 15)
Age, years (Mean ±SD)		51.8 (10.6)	47.0 (9.2)	50.9 (9.08)
Gender	Male	7 (46.7)	8 (53.3)	9 (60.0%)
	Female	8 (53.3)	7 (46.7)	6 (40.0%)
Race	Malay	10 (66.7)	10 (66.7)	13 (86.7)
	Chinese	5 (33.3)	4 (26.7)	2 (13.3)
	Others	1 (6.7)	1 (6.7)	0 (0.0)

The means and standard deviation of pocket depth in the tetracycline group (5.22 ±0.27 mm), minocycline (5.51±0.45 mm), and control group (5.36±0.44mm) were similar at the beginning of the study (P > 0.05) (Table 2). Repeated measures ANOVA were carried out to examine the differences in pocket depth changes between the tetracycline, minocycline, and control groups after one month, between one month and three months, and over the course of three months. Mauchly’s test indicated that the sphericity assumption was met (P > 0.05). The results of the analysis showed that after one month, the greatest mean pocket depth changes were seen in the tetracycline group (mean changes = 1.4 mm, P < 0.001), followed by the control group (mean changes = 1.0 mm, P < 0.001) and the minocycline group (mean changes = 1.0 mm, P < 0.001). Between one month and three months, all the groups showed significant differences in pocket depth changes except the control group (P >0.05). Pocket depth changes were statistically significant in all three groups (P < 0.001) over the three months of treatment, with the tetracycline group showing the greatest mean pocket change (mean changes = 1.79 mm, P < 0.001). Within-group observation showed highly significant (P < 0.001) changes in probing depth from baseline to three months in all three study groups except between one month and three months in the control group. An analysis between groups showed that there was no significant difference in the periodontal pocket means between the tetracycline, minocycline, and control groups (F test = 2.3, P > 0.05) (Table 4). There was also no significant difference in mean pocket depth among the three study groups based on time (F test = 1.153, P > 0.05).

**Table 2:** Comparison of periodontal pocket depth among three groups.

Variables		Tetracycline	Minocycline	Control	P- value
Pocket depth (mm) (Mean±SD)	Baseline	5.22 (0.266)	5.51 (0.445)	5.36 (0.438)	0.1
	1 month	3.78 (0.752)	4.53 (0.838)	4.35 (1.072)	-
	3 months	3.42 (0.725)	4.05 (0.984)	3.62 (1.357)	-
Pocket depth changes (mm) (95%CI) P- value	Changes at 1 month	1.44 (0.976, 1.900) <0.001	0.99 (0.570, 1.406) <0.001	1.01 (0.515, 1.505) <0.001	0.1
	Changes between 1 month and 3 months	0.36 (0.019, 0.690) 0.037	0.48 (0.086, 0.866) 0.015	0.73 (-0.016, 1.481) 0.1	
	Overall Changes	1.79 (1.361, 2.225) <0.001	1.46 (0.975, 1.953) <0.001	1.74 (0.923, 2.562) <0.001	

**Figure 2:**
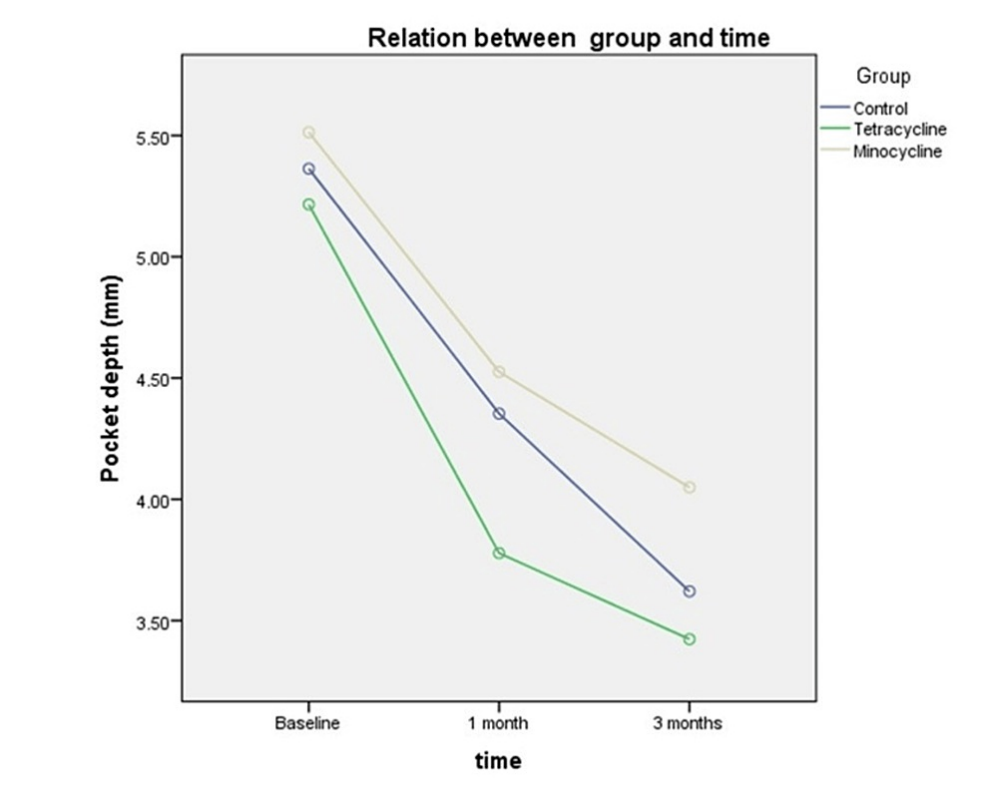
Comparison of mean pocket depth among three different groups based on time.

Figure 2 shows a steep slope between the start of the treatment and the one-month review in all treatment groups, but it flattened between the one-month and three-month reviews. The tetracycline group showed greater pocket depth changes compared to the other groups one month after the treatment, while the control group showed a steady reduction throughout the three-month period, and the minocycline group showed the least.

Meanwhile, the mean clinical attachment loss (CAL) in the tetracycline group (3.06±0.85mm), minocycline (3.05±1.17mm), and control group (3.61±1.69mm) at the commencement of the study were similar, with the control group showing the highest clinical attachment loss (P > 0.05) (Table 3). Repeated measures ANOVA were carried out to examine the differences in clinical attachment loss changes between the tetracycline, minocycline, and control groups after one month, between one month and three months, and over the course of three months. Mauchly’s test indicated that the sphericity assumption was not met (P < 0.05). Within-group analysis showed that there was a significant reduction in clinical attachment loss in all treatment groups after intervention at one month and three months, except between one month and three months, in the tetracycline (P >0.05) and minocycline groups (P > 0.05). After one month, the highest improvement in clinical level was seen in the minocycline group (mean changes = 0.7mm, P < 0.05) and the lowest in the tetracycline group (mean changes = 0.6mm, P <0.05). The overall improvement in attachment level was seen in the control group (mean changes = 1.1mm, P < 0.05), followed by the minocycline group (mean changes = 1.0 mm, P <0.05). The analysis between groups showed that there was no difference in the changes in clinical attachment loss between the tetracycline, minocycline, and control groups (F test = 0.498, P > 0.05). Additionally, there was no significant difference in mean clinical attachment loss among the three study groups based on time (F test =0.166, P > 0.05).

**Table 3:** Comparison of clinical attachment loss among three groups.

Variables		Tetracycline	Minocycline	Control	P-value
CAL (mm) Mean (SD)	Baseline	3.06 (0.850)	3.05 (1.172)	3.61 (1.690)	0.4
	1month	2.42 (1.321)	2.34 (1.727)	2.95 (2.043)	-
	3 months	2.19 (1.624)	2.10 (1.817)	2.51 (2.262)	-
CAL changes (mm) Mean (95%CI) P-value	Variables	Tetracycline	Minocycline	Control	0.6
	Changes at 1 month	0.6 (0.088,1.179) 0.021	0.71 (0.172,1.239) 0.009	0.65 (-0.003,1.311) 0.051	
	Changes between 1 month & 3 months	0.24 (-0.250,0.729) 0.6	0.25 (-0.387,0.884) 1.0	0.44 (0.034,0.846) 0.032	
	Overall Changes	0.87 (0.045,1.701) 0.038	0.95 (0.203,1.705) 0.012	1.10 (0.394,1.794) 0.002	

**Figure 3:**
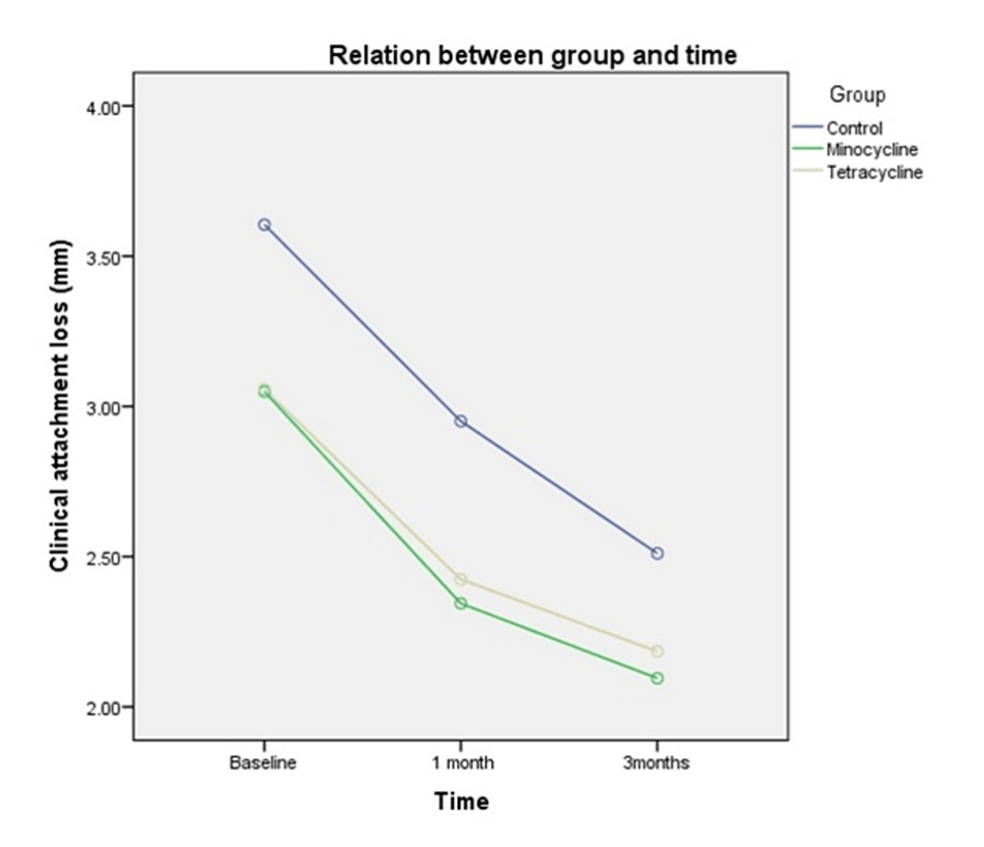
Comparison of mean clinical attachment loss among three different groups based on time.

Figure 3 shows a steep slope of the lines between the start of the treatment and the one-month review in all treatment groups but flattened between the one-month and three-month review. The tetracycline and minocycline groups showed high clinical attachment loss compared to the control group over the course of three months. The control group showed the least clinical attachment loss.

Moreover, bleeding on probing percentage in the tetracycline group (52.51±21.79%), minocycline group (55.87±14.15%), and control group (40.63±19.41%) were similar at the initial part of the study (p > 0.05). A repeated measure ANOVA was carried out to examine the differences in the means of bleeding on probing percentage between the tetracycline, minocycline, and control groups after one month, between one month and three months, and the overall changes over the three months. Mauchly’s test indicated that the sphericity assumption was not met (p < 0.001). Results of analysis showed that, after one month of treatment, the greatest changes in bleeding on probing percentage were observed in the minocycline group (mean changes = 19.34%, p < 0.001), followed by the tetracycline group (mean changes = 10.43%, p < 0.05), and the control group (mean changes = 9.09%, p < 0.05). Bleeding on probing percentage over the three months was also found to be statistically significant in all three treatment groups, with the minocycline group showing the greatest mean changes (mean changes = 26.42%, p < 0.001) (Table 4). The analysis also showed that there was no significant difference in bleeding on probing percentage between the tetracycline, minocycline, and control groups (F test = 2.123, p > 0.05). The analysis showed that there was no significant difference in bleeding on probing percentage among the three study groups based on time (F test = 2.598, p > 0.05). 

**Table 4:** Comparison in bleeding on probing percentage among three groups.

Variables		Tetracycline	Minocycline	Control	P-value
Bleeding on Probing (%) Mean (SD)	Baseline	52.53 (21.790)	55.87 (14.150)	40.63 (19.414)	0.1 -
	1 month	42.10(13.943)	36.53(12.884)	31.54(14.537)	
	3 months	30.38(6.657)	29.45(10.599)	25.87(13.905)	
Bleeding on Probing (%) changes (95%CI) P-value	Variables	Tetracycline	Minocycline	Control	0.1
	Changes at 1 month	10.43 (1.844,19.016) 0.016	19.34 (13.952,24.722) 0.000	9.09 (0.803,17.371) 0.030	
	Changes between 1 month & 3 months	11.72 (3.216,20.217) 0.007	7.09 (0.185,13.989) 0.043	5.67 (1.088,10.248) 0.014	
	Overall Changes	22.15 (8.503,35.790) 0.002	26.42 (18.374,34.473) 0.000	14.76 (5.584,23.925) 0.002	

**Figure 4:**
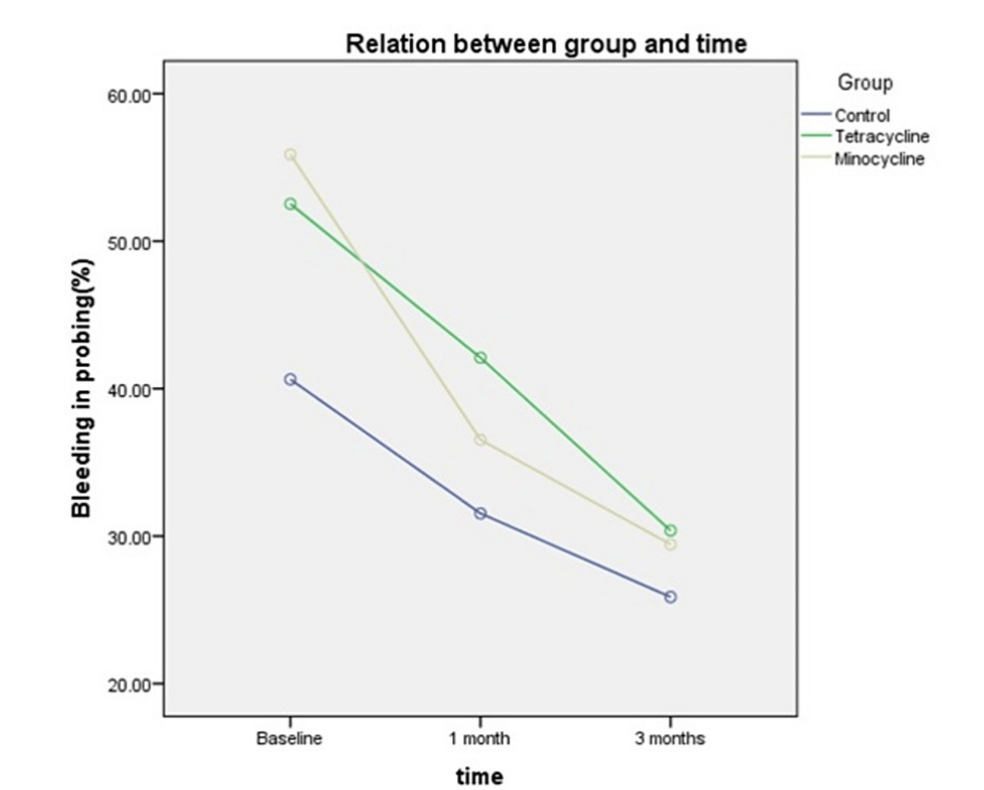
Comparison of mean bleeding in probing among three study groups on time.

Figure 4 shows that the minocycline group had the steepest slope between the start of treatment and the one-month review compared to the tetracycline and control groups and the least steep slope between one month and three months. Tetracycline and control groups showed steady changes in the bleeding on probing percentage over the course of three months.

## Discussion

Probing pocket depth and clinical attachment loss are the main parameters used for the assessment of treatment outcomes for periodontitis. SRP is an important procedure in non-surgical periodontal therapy (NSPT) to halt disease progression and eliminate dental biofilm in the deep periodontal pocket.

Periodontal diseases caused by bacteria plaque cause local inflammation and destructive bone loss within the periodontium [16]. Adjunctively, locally delivered antibiotics for periodontal disease are often used to reduce the microorganisms found within the oral cavity, improve the healing of periodontium, and inhibit the recurrence of inflammation [17]. A previous study by Pandit et al. (2013) reported there was a significant decrease in probing pocket depth and gain in clinical attachment level at one and three months in all treatment groups when compared to baseline (P<0.001) [18]. Thus, the local antibiotic minocycline has been chosen as our target antibiotic, and we have analyzed its effect on periodontal diseases since it has a lower risk of developing resistance against *A. actinomycetemcomitans*, *T. forsythia* and *P. gingivalis* [19]. Moreover, minocycline was also proven to have anti-inflammatory, immunomodulatory, and antioxidant properties [20].

In this present study, all groups demonstrated significant changes for PD and CAL after a three-month re-evaluation, although the changes from one to three months were not significant for PD in SRP alone or for CAL in SRP that received antimicrobial treatment. The highest changes for PD were seen in the group receiving tetracycline (1.79mm) and in the SRP-only group for CAL (1.1mm). Nevertheless, the mean changes of these parameters showed no significant difference among the three groups. In a systematic review conducted by Heitz-Mayfield et al. (2002), NSPT was reported to give 0.4 mm more clinical attachment gain and 0.4 mm less probing depth reduction than surgical therapy in pockets of 4 to 6 mm [21].

Both groups were shown to significantly reduce all the clinical parameters after 1 month. A baseline comparison of clinical parameters between groups at pre-treatment was conducted to omit any bias. The mean changes in the SRP+M group compared with the SRP group were 1.75 (0.93) and 1.92 (0.84) for pocket depth and 2 (1.48) and 2.11 (0.63) for CAL, respectively. This might be due to the fair effectiveness of locally delivered minocycline as an adjunct to SRP over a short study period. Even though topically applied antibiotics have been reported to show some benefit compared to SRP alone, the results were shown to be modest and mostly short-term. Moderate improvements in probing depth reductions compared with SRP alone were shown in a systematic review [22]. Overall, the inflammation showed significant improvement, as reflected by a reduction in bleeding on probing after three months in all groups, with higher mean changes in groups receiving tetracycline and minocycline, although the mean changes showed no significant difference between groups. Reduced bleeding on probing demonstrated resolution of inflammation as a result of periodontal healing after NSPT [22].

Performing effective SRP would be enhanced by the appropriate instrument used. Powered instruments have gained popularity with time, owing to their increasing efficacy and efficiency in performing SRP. More recently, these power-driven instruments have been modified to have smaller-diameter tips and longer working lengths, thus providing better access to deep probing sites and more efficient subgingival instrumentation [23].

Gracey curettes were also used for root planing during our study, as there were no significant differences reported in the clinical and microbiological outcomes achieved between powered instruments and hand instruments [23,24]. However, initial pocket depth, operator experience, tooth type, and surface were found to influence the effectiveness of calculus removal [24]. Restricted access to furcation areas, deep pockets, and irregular surfaces complicates the removal of bacterial deposits; thus, the procedure is vastly dependent upon the skills of the clinician [25].

## Conclusions

Local application of minocycline and tetracycline as adjuncts to SRP has the potential to enhance the healing result, known as a reduction in bleeding on probing and pocket depth, as well as a gain in clinical attachment level. However, it does not offer an advantage over the conventional method of SRP alone. The main limitation of this study is the small number of samples and the short observation period. Further study with a larger sample size, longer follow-up duration, and confirmation with the microbiological analysis is recommended in the future to overcome the drawbacks of the present study.
